# Mast Cell-Mediated Inhibition of Abdominal Neutrophil Inflammation by a PEGylated TLR7 Ligand

**DOI:** 10.1155/2012/262394

**Published:** 2011-11-17

**Authors:** Tomoko Hayashi, Shiyin Yao, Brian Crain, Michael Chan, Howard B. Cottam, Fitzgerald Lao, Dennis A. Carson, Maripat Corr

**Affiliations:** ^1^Moores Cancer Center, University of California San Diego, 3855 Health Sciences Drive, La Jolla, San Diego, CA 92093-0820, USA; ^2^Department of Medicine, University of California San Diego, 9500 Gilman Drive, La Jolla, San Diego, CA 92093-0663, USA

## Abstract

Although the mechanisms for sustained chemokine gradients and recurring cell infiltration in sterile peritonitis have not been elucidated, toll-like receptors (TLRs) have been implicated. To abate the deleterious recruitment of neutrophils in sterile inflammation, we repeatedly administered a TLR7 ligand that hyposensitized to TLR7 and receptors that converged on the MyD88-signaling intermediary and reduced cellular infiltration in murine autoimmune models of multiple sclerosis and arthritis. To reduce potential adverse effects, a polyethylene glycol polymer was covalently attached to the parent compound (Tolerimod1). The proinflammatory potency of Tolerimod1 was 10-fold less than the unconjugated TLR7 ligand, and Tolerimod1 reduced neutrophil recruitment in chemically induced peritonitis and colitis. The effects of Tolerimod1 were mediated by the radioresistant cells in radiation chimeric mice and by mast cells in reconstituted mast cell-deficient mice (*Kit* ^*W*-*sh*^). Although the Tolerimod1 had weak proinflammatory agonist activity, it effectively reduced neutrophil recruitment in sterile peritoneal inflammation.

## 1. Introduction

The inflammatory response is a major host defense mechanism to prevent infection or to repair injury [1]. However, in the context of sterile inflammation the perpetual recruitment of neutrophils into the local environment can lead to deleterious sequelae from proteases and other products released from their granules [2]. The early events of inflammation include increased vascular permeability and enhanced immune cell mobility to allow cells and proteins to access the site of inflammation [3]. Chronicity is established when sustained levels of chemokines attract the influx of neutrophils and monocytes. Early disruption of this recurring cycle and reduction of neutrophil recruitment could suppress subsequent infiltration of other types of immune cells and could then prevent tissue damage [4].

The myeloid differentiation primary response gene 88-(MyD88) signaling pathway has been implicated in perpetuating the inflammatory response in experimental peritonitis and colitis. This pathway is shared by the TLRs except TLR3, which uses the MyD88-independent TIR-domain-containing adapter-inducing interferon-*β* (TRIF) pathway [5]. In a previous study, we demonstrated that chronic administration of low doses of a synthetic TLR7 ligand (9-benzyl-8-hydroxy-2-(2-methoxyethoxy) adenine; designated here as 1V136) provided pharmacological suppression of the MyD88-signaling pathway and subsequently reduced the severity of inflammation in mouse models of autoimmunity [6].

We have synthesized modifications of 1V136 to study the effects on potency and alter the pharmacodynamics to reduce potential toxicities [7]. One compound that was modified with a 6 unit PEG moiety had reduced ability to induce inflammatory cytokines *in vitro* and *in vivo* and was designated Tolerimod1 [7]. TLR-mediated signals can be ultimately protective in models of acute intestinal inflammation and considered to be therapeutic targets [8, 9]. Hence Tolerimod1 was selected to test our hyposensitization strategy to limit inflammatory recruitment in mouse models of peritonitis and inflammatory bowel disease.

 In the present study, the hyposensitization effect of repeated dosage with the parent compound 1V136 was confirmed in the murine acute dextran sodium sulfate (DSS)-colitis model. The anti-inflammatory effects of Tolerimod1 were also tested in the DSS-colitis and thioglycolate (TG)-elicited peritonitis models. The PEGylated compound was equivalent to the parent compound in its ability to reduce peritoneal inflammation and DSS-colitis. Radiation chimeras using wild-type (WT) and *Tlr*7^−/−^ mice indicated that this effect was mediated by the radioresistant cells in the recipient and not by the transferred bone marrow cells. In addition mast cell-deficient mice (*Kit* ^*W*-*sh*^) were relatively refractory to the therapeutic treatment. However, the treatment response was recapitulated in mice that had been reconstituted with bone marrow cells enriched for mast cells. These results implicate mast cells as a primary effector cell for Tolerimod1 activity *in vivo*.

## 2. Materials and Methods

### 2.1. Animals

C57BL/6 (wild type, WT) and *Kit* ^*W*-*sh*^/HNihrJaeBsmJ mice were purchased from the Jackson Laboratories (Bar Harbor, MA, USA). *Tlr*7^−/−^ mice were a gift from Dr. Akira (Osaka University, Japan) and backcrossed for 10 generations onto the C57BL/6 background at University of California, San Diego (UCSD). Bone marrow chimeras were generated by injecting 10^7^ bone marrow cells intravenously into whole-body irradiated (950 cGy) recipient mice [6]. *Kit* ^*W*-*sh*^ mice were engrafted with mast cells by intravenous (i.v.) injection of 10^6^ mast cells as previously described [10]. All animal experiments were approved by the UCSD Institutional Animal Care and Use Committee.

### 2.2. Reagents

Dextran sodium sulfate (DSS) was purchased from Sigma (St Louis, MO). Thioglycolate (TG) medium, PBS, RPMI 1640 medium, and DMEM were purchased from Invitrogen (Carlsbad, CA, USA). RPMI 1640 and DMEM were supplemented with 10% FCS, 100 U/mL penicillin, and 100 *μ*g/mL streptomycin. 1V136 (unconjugated TLR7 ligand) and PEGylated TLR7 ligand (Tolerimod1, TMX-302, described as compound 1b in [7]) were prepared in our laboratory as previously described [7, 11]. 1V136 and Tolerimod1 were dissolved in DMSO as a 100 mM stock solution and kept at −20°C until use. Endotoxin levels in all reagents were measured using Endosafe (Charles River laboratory, Wilmington, MA, USA). Endotoxin levels of the compounds were <0.05 EU/*μ*mol.

### 2.3. Compound Activity Tests

The RAW264.7 mouse monocyte macrophage cell line was obtained from ATCC (Rockville, MD, USA) and cultured in DMEM (Irvine Scientific, Irvine, CA, USA) supplemented with 10% heat-inactivated fetal bovine serum, 2 mM L-glutamine, and 100 U/mL penicillin/100 *μ*g/mL streptomycin). Primary bone marrow-derived dendritic cells (BMDCs) were prepared from C57BL/6 mice as described [12]. The cells (1 × 10^6^/mL) were incubated with the indicated compound for 18 hours at 37°C, 5% CO_2_, and culture supernatants were collected. IL-6, IL-10, IL-1*β*, TNF*α*, and KC were measured from the supernatants or sera by ELISA (BD Bioscience, or eBioscience, San Diego CA, USA). Minimum detection levels of these factors ranged from 5 to 15 pg/mL.

### 2.4. Induction of DSS-Colitis

WT mice were given 2% (wt/vol) DSS dissolved in sterilized, distilled water *ad libitum* for 7 days. Mice were injected subcutaneously (subcutaneously) with daily doses of 1V136 (150 nmol), Tolerimod1 (200 nmol) or vehicle on days 0 to 7. On day 8, mice were sacrificed and disease activity index (DAI; the combined score of weight loss and intestinal bleeding) was determined as described previously [13].

### 2.5. Induction of TG-Induced Peritonitis

Mice were injected s.c. with a single dose (day-1 or -3) or three daily doses of 1V136 (150 nmol) or Tolerimod1 (200 nmol). On day 0, mice were intraperitoneally (i.p.) injected with 2 mL 4% TG medium. Peritoneal exudates were collected at 3 h with 3 mL cold PBS. Total cell number was determined by counting with a hematocytometer. Peritoneal cells were cyto-centrifuged and stained with Wright-Giemsa to determine differential leukocyte counts.

### 2.6. Histological Analysis

Colons were fixed in buffered formalin and embedded in paraffin. Five *μ*m sections were cut and stained with hematoxylin and eosin (H&E). The sections were stained for myeloperoxidase (MPO) using rabbit anti-MPO antibody (Abcam, Cambridge, MA) and horseradish peroxidase-(HRP) conjugated anti-rabbit IgG (Jackson ImmunoResearch, West Grove, PA, USA) by the Histology and Immunohistochemistry Shared Resources at the Moores UCSD Cancer Center.

### 2.7. Myeloperoxidase (MPO) Assay

MPO activity (kinetic assay) was performed as previously described with minor modifications [14]. Briefly, colon tissues were homogenized in 0.5% hexadecyltrimethyl-ammonium bromide (Sigma) in 50 mM phosphate buffer, pH 6.0. The homogenate was sonicated for 10 seconds, frozen and thawed 3 times, and then centrifuged for 15 minutes. Protein concentration in the supernatants was quantified by bicinchoninic acid assay (Thermo Scientific Pierce). The supernatants were diluted 1 : 30 in reaction buffer (0.68 mM O-dianisidine, 50 mM potassium phosphate buffer, pH 6.0, 29 mM H_2_O_2_), and MPO activity was measured as the absorbance at 460 nm during the first 2 minutes, and expressed as OD per minute per mg of protein.

### 2.8. Assessment of Vascular Permeability

Briefly, mice were intravenously injected with 200 *μ*L 0.625% Evans blue solution 10 min before TG injection. After two hours peritoneal lavage was performed using 2 mL cold PBS, and absorbance at 620 nm was measured.

### 2.9. Statistical Analysis

The data are represented as means ± standard errors of the mean (SEM). The Mann-Whitney test was used to compare two groups, and one-way ANOVA with Dunnett's* post hoc* test was used for multiple comparisons to a control group using Prism 4 (GraphPad Software, San Diego, CA, USA).

## 3. Results

### 3.1. Repeated Treatment with a TLR7 Ligand-Reduced Disease Severity in DSS-Colitis

**Figure 1:**
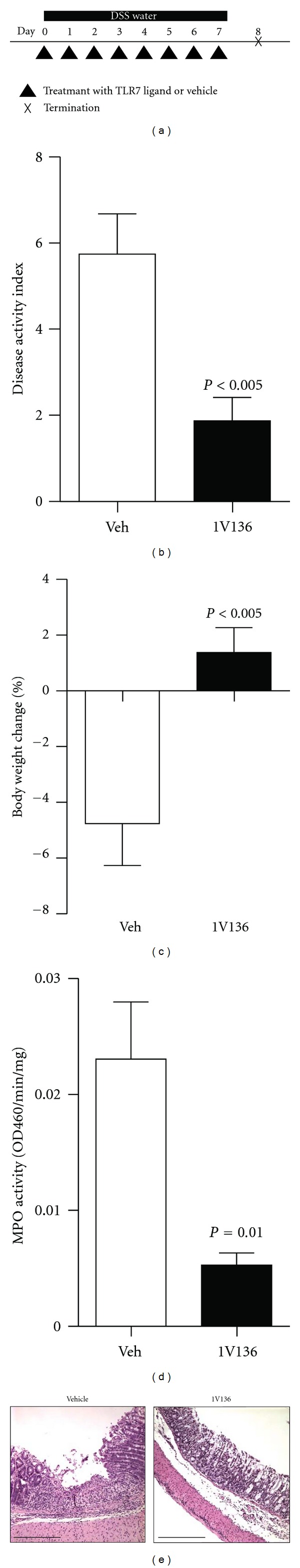
TLR7 ligand hyposensitization reduces the inflammation of DSS-induced experimental colitis. (a) Experimental protocol of induction of DSS-induced colitis and treatment regimen. WT mice (*n* = 5) received 2% DSS for 7 days. Mice were s.c. treated with 150 nmol 1V136 or vehicle. (b) Disease activity index, (c) percent body weight changes, and (d) MPO activity of colonic tissue was determined as described in Section 2. Data are expressed as means ± SEM and are representative of 2 independent experiments. *Denotes *P* < 0.05 by Mann-Whitney test. (d) Examples of colons of vehicle- or 1V136-treated colitis mice that were removed and prepared for histological examination. Original magnification is ×100. Calibration bar: 100 *μ*m.

We previously demonstrated that the repeated administration of a TLR7 ligand ameliorated joint inflammation in the K/BxN serum-transferred arthritis model and reduced inflammatory cell influx in the EAE model [6]. To examine this effect on sterile abdominal inflammation, we tested this treatment regimen in the DSS-colitis model. Mice were treated daily with 150 nmol s.c. 1V136 (unconjugated TLR7 ligand) starting on day 0 until the end of the experiment (Figure 1(a)). This was the lowest s.c. dosage that was effective when administered s.c. in the K/BxN serum transferred arthritis model [6]. Mice treated with daily 1V136 exhibited significantly lower DAI (*P* = 0.005, Figure 1(b)) and less body weight loss (*P* = 0.005, Figure 1(c)). Decreased disease severity was concordant with decreased MPO activity in the colons of 1V136-treated mice (Figure 1(d)). Histological examination showed fewer ulcerative lesions and less inflammatory cell infiltration in the colons of 1V136-treated mice compared to vehicle-treated mice (Figure 1(e)).

### 3.2. Tolerimod1 Reduced Neutrophil Recruitment in TG-Elicited Peritonitis

**Figure 2:**
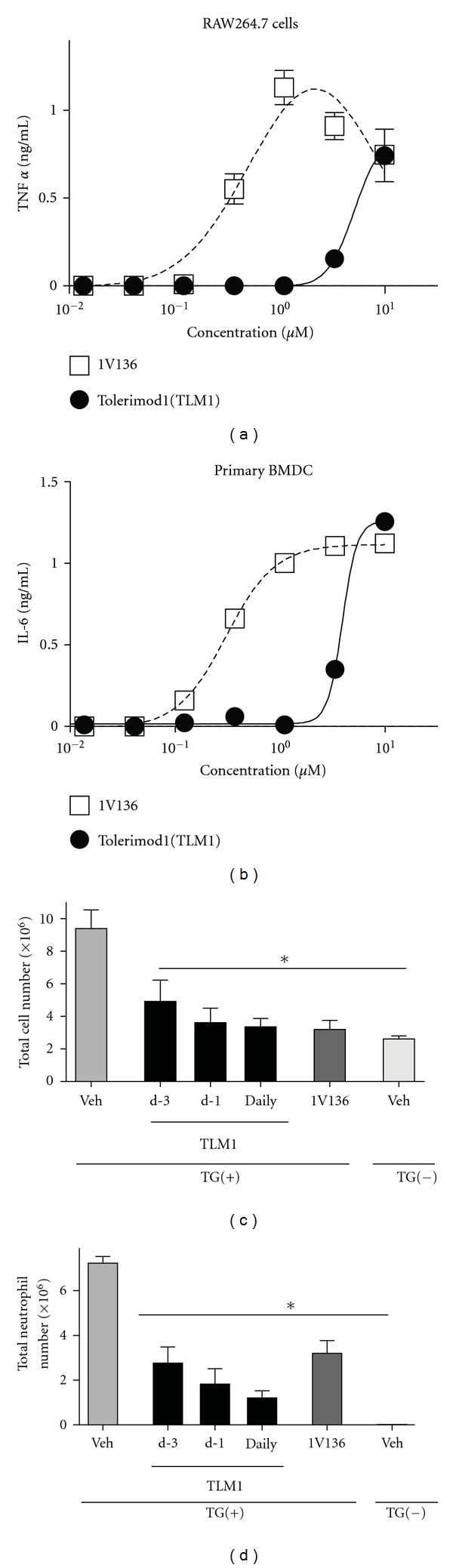
PEGylated TLR7 ligand reduced neutrophil recruitment in TG-elicited peritonitis. RAW264.7 (a) and primary BMDC (b) were treated 18 h with Tolerimod1 (TLM1), and TNF*α* and IL-6 in the supernatants were measured by ELISA. (c and d) WT mice (*n* = 7 to 10 per group) were treated daily for three days (-3, -2, and -1) or one day (-3 or -1 day) of s.c. 200 nmol Tolerimod1 (TLM1). Peritonitis was induced on day 0. Total cell number (c) and total neutrophil number (d) recovered in the peritoneal lavage were measured. Data shown are mean ± SEM and are pooled 2 independent experiments that showed similar trends. *Denotes *P* < 0.05 by one way ANOVA with Dunnett's *post hoc* testing compared to the vehicle-treated group.

In a previous study, we observed that a higher dose (500 nmol) of 1V136 administered intraperitoneally or intranasally resulted in TLR7-dependent adverse effects [10]. To limit these adverse effects, we prepared several modified forms of the parent compound and tested them *in vitro* and *in vivo* for chemokine and cytokine release [7]. Amongst these compounds, one containing a 6-unit PEG chain, was a weakly active TLR7 agonist (Tolerimod1). Dose titration studies are shown with a murine macrophage cell line, RAW264.7 cells, and primary murine BMDCs stimulated with Tolerimod1 and 1V136 for cytokine release. Tolerimod1 (EC50_RAW_ = 4.8 *μ*M and EC50_BMDC_ = 3.9 *μ*M) was 10 times less potent than 1V136 (EC50_RAW_ = 0.37 *μ*M and EC50_BMDC_ = 0.3 *μ*M) (Figures 2(a) and 2(b)).

 The unconjugated parent TLR7 ligand (1V136) reduced MPO-positive cell infiltration in DSS-colitis (Figure 1(d)), indicating that 1V136 treatments attenuated neutrophil recruitment into the colon. To further examine the mechanism of Tolerimod1 effects on neutrophil recruitment, Tolerimod1 was used in the TG-elicited peritonitis model (Figures 2(c) and 2(d)). Mice received three daily treatments (days-3, -2, and -1), or a single treatment on day-1 or day-3 of Tolerimod1, and peritonitis was induced on day 0. Tolerimod1 treatment reduced overall cell infiltration (Figure 2(c)), primarily neutrophil influx (Figure 2(d)) into the peritoneal cavity. In addition mice that were pretreated with daily s.c. (200 nmol) or oral (200 nmol) Tolerimod1 for three days and then were injected with TG intraperitoneally had a significant reduction in total neutrophil count after 3 h (2.33 ± 0.33 × 10^6∗^, and 2.39 ± 0.38 × 10^6∗^, respectively, compared to 4.60 ± 0.51 × 10^6^ in the vehicle control; *P < 0.05 by one-way ANOVA). However, the number of infiltrating monocytes after 3 days daily s.c. (200 nmol) of pretreatment was not significantly reduced (2.43 ± 0.30 × 10^6^ versus 3.1 ± 0.5 × 10^6^ in the vehicle control).

### 3.3. Reduction in Peritoneal Neutrophil Influx Was Associated with Reduced Local Chemokine Levels

**Figure 3:**
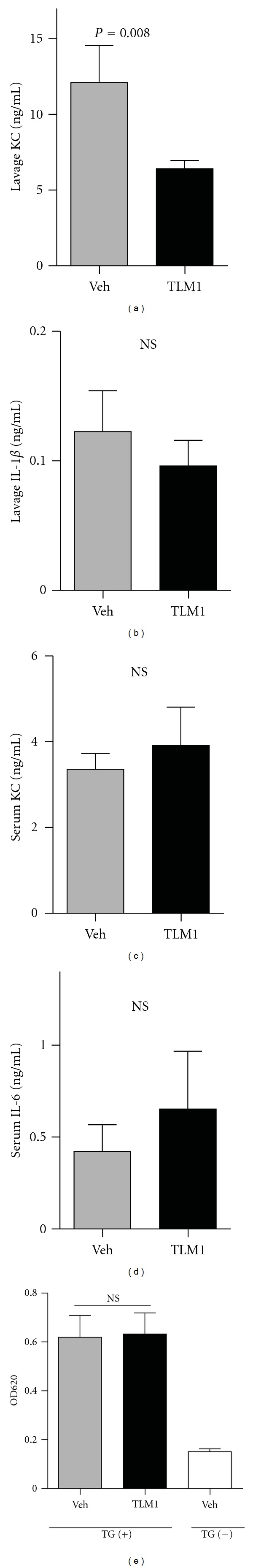
Tolerimod1 treatment reduced local chemokine release but had no effect on systemic proinflammatory cytokine levels. TG-elicited peritonitis was induced in Tolerimod1 (TLM1) or vehicle (veh)-treated mice (*n* = 8–10 per group). Sera and peritoneal lavage were harvested 3 h after TG injection. Levels of KC (a) and IL-1*β* (b) in the lavage and KC (c) and IL-6 (d) in sera were measured by ELISA. IL-1*β*, TNF*α*, and IL-10 in the sera or lavage, or IL-6 in the lavage, were below detection levels. (e) Vascular permeability was measured by Evans blue assay. Peritoneal lavage was collected 2 h after TG injection. Data shown are mean ± SEM and are pooled from 2 independent experiments that showed similar trends. *P* values are compared between vehicle (veh)- and Tolerimod1 (TLM1)-treated group by Mann-Whitney test. NS indicates not significant.

The immune cell recruitment is influenced by numerous factors, such as local chemokine secretion and vascular permeability. Hence, we examined the levels of chemokines and cytokines in sera and peritoneal lavages 3 h after TG peritonitis induction. Tolerimod1 treatment significantly reduced KC levels in the lavage (Figure 3(a)), but IL-1*β* levels were not reduced (Figure 3(b)). In contrast, serum levels of KC and IL-6 were not influenced by Tolerimod1 treatments (Figures 3(c) and 3(d)). IL-1*β*, TNF*α*, and IL-10 in the sera and peritoneal lavage and IL-6 in the peritoneal lavage were below detectable levels (data not shown). Tolerimod1 treatment did not alter the vascular permeability as measured by Evans blue dye extravasation (Figure 3(e)).

### 3.4. Radioresistant Mast Cells Were Involved in Anti-Inflammatory Effects of Tolerimod1

**Figure 4:**
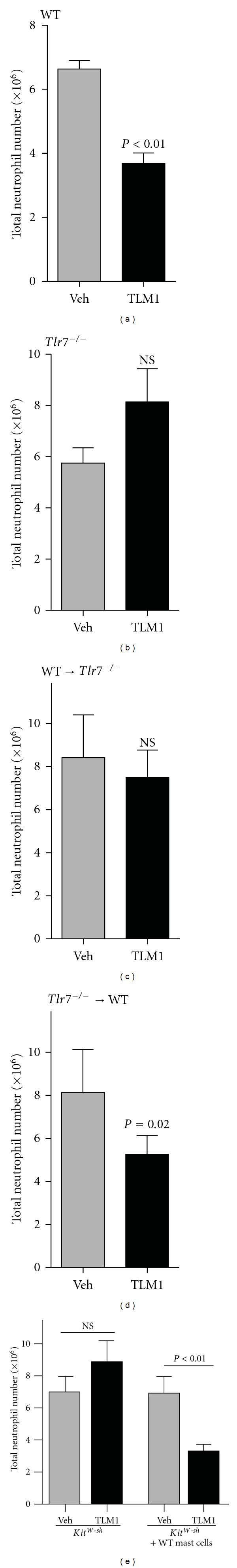
Radioresistant mast cells were involved in suppression of neutrophil recruitment by Tolerimod1 treatment. (a) WT, (b) *Tlr*7^−/−^, or chimeric mice [WT→ *Tlr*7^−/−^ (c), and *Tlr*7^−/−^→WT (d)] were treated with 200 nmol Tolerimod1 (TLM1) s.c. for 3 days and peritonitis was induced with TG (*n* = 4 to 8 per group). (e) Mast cell-deficient *Kit* ^*W*-*sh*^ mice or *Kit* ^*W*-*sh*^ mice reconstituted with WT mast cells were treated with 200 nmol Tolerimod1 (TLM1) s.c. for 3 days, and peritonitis was induced with i.p. TG. The peritoneal cells were recovered by lavage after 3 hours and quantitated. Data shown are mean ± SEM and are pooled from 2 independent experiments that showed similar trends. *P* values are from comparisons of vehicle (veh)- and Tolerimod1-treated groups by Mann-Whitney test.

Since Tolerimod1 treatment influences local chemokine levels, but not systemic levels of chemokines or cytokines, we thought it important to identify the cell types in the local tissue involved in the effects of Tolerimod1. Tolerimod1 treatment in *Tlr*7^−/−^ mice did not reduce TG-elicited recruitment of inflammatory cells (Figures 4(a) and 4(b)), indicating that the treatment effects of Tolerimod1 were TLR7 dependent. To further test the involvement of hematopoietic cells in the effects of Tolerimod1 treatment, *Tlr*7^−/−^ and WT (C57BL/6) radiation bone marrow chimeric mice were generated. Interestingly, reduced peritoneal neutrophil recruitment after TG injection was observed in the WT donor → *Tlr*7^−/−^ recipients, but not in *Tlr*7^−/−^ donor→WT recipients (Figures 4(c) and 4(d)), suggesting that the radioresistant cell population was predominantly mediating the anti-inflammatory effects of Tolerimod1.

 Mast cells are relatively radioresistant and involved in many inflammatory processes associated with an increase in vascular permeability [15–17]. To examine the potential role of mast cells, mast cell-deficient *Kit* ^*W*-*sh*^ mice were treated with Tolerimod1 for 3 days, and peritonitis was induced. Tolerimod1 treatment did not reduce neutrophil infiltration into the peritoneum of *Kit* ^*W*-*sh*^ mice (Figure 4(e)). Furthermore, the response to Tolerimod1 treatment in *Kit* ^*W*-*sh*^ mice was restored by reconstitution of WT mast cells into *Kit* ^*W*-*sh*^ mice (Figure 4(e)). Of note is the relative neutrophilia in *Kit* ^*W*-*sh*^ mice, which appeared as a trend in the higher neutrophil recruitment in the mast cell replete mice [18].

### 3.5. Tolerimod1 Reduced Severity of DSS-Colitis

**Figure 5:**
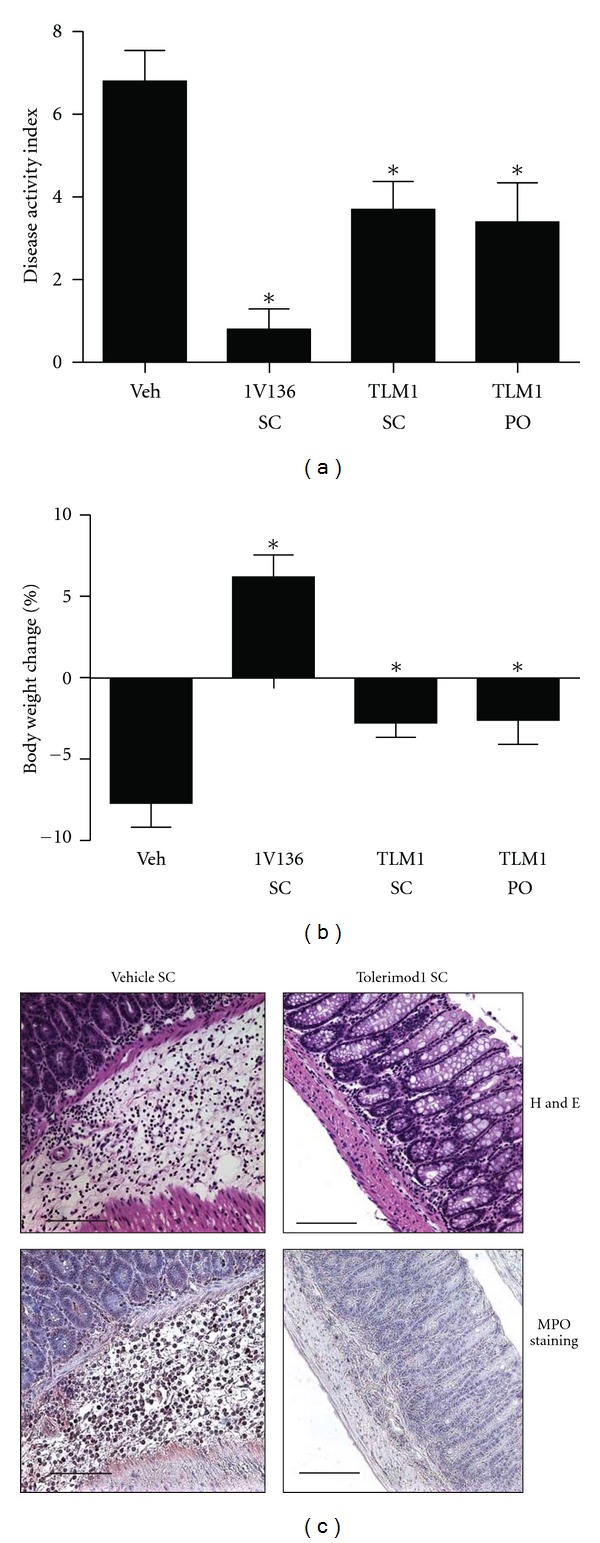
Tolerimod1 reduced the severity of DSS-induced colitis (a and b) DSS-colitis mice (*n* = 12 per group) were treated with 200 nmol Tolerimod1 subcutaneously (SC) or orally (PO) for 7 days. Disease activity index (a) and body weight loss (b) were evaluated. (c) The colons isolated from mice treated s.c. with vehicle or Tolerimod1 were stained with hematoxylin or eosin, or immunostained for MPO. Data shown are mean ± SEM and are pooled from 2 independent experiments that showed similar trends. *Denotes *P* < 0.05 by one-way ANOVA with Dunnett's *post hoc* testing compared to the vehicle-treated group.

Repeated administration of unconjugated TLR7 ligand, 1V136, reduced neutrophil inflammation in DSS-colitis (Figure 1). Our data indicate that the inhibitory effect of Tolerimod1 on neutrophil infiltration was at least in part mediated by mast cells, which are known to be involved in intestinal inflammation in DSS-colitis [19]. Hence, we treated DSS-colitis mice with Tolerimod1 s.c. or orally with daily Tolerimod1 for a week (Figure 1(a)). Both s.c. and oral administration of Tolerimod1 reduced the DAI and prevented body weight loss (Figures 5(a) and 5(b)). Histologic examination revealed a reduction in inflammatory cell infiltration in the lamina propria (Figure 5(c)). There was also a notable reduction in staining for MPO-positive neutrophils (Figure 5(c)).

## 4. Discussion

Neutrophils are the first responders in acute injury and inflammation. During the early phase of inflammation, neutrophils migrate from blood vessels to the site of inflammation. In the tissue, neutrophils can then mediate damage by releasing the contents of their granules and further amplifying inflammatory processes [1]. We previously demonstrated that repeated administration of an unconjugated TLR7 ligand (1V136) reduced joint inflammation in the neutrophil-dependent K/BxN serum transfer arthritis model [6]. In the current study, we tested the anti-inflammatory effects of TLR7 ligands in the TG peritonitis and DSS-colitis models. Neutrophil-associated inflammation was markedly attenuated by oral or subcutaneous administration of doses of the parent compound, 1V136, or a PEG-modified version, Tolerimod1 [7]. Although proinflammatory potencies of PEGylated TLR7 ligand (Tolerimod1) was 10 times less than unconjugated TLR7 ligand, it retained anti-inflammatory properties in DSS-colitis as well as TG-elicited peritonitis models.

Mucosal administration of 1V136 resulted in hypothermia and caused anorexic behavior that was associated with TNF*α* release [10]. Hence, we hypothesized that the adverse effects could be attenuated by reducing the proinflammatory potency of the drug. Among the PEGylated derivatives of 1V136 that we prepared, we found that a conjugate with a 6-unit PEG chain (Tolerimod1 in this study) had minimal TLR7-dependent proinflammatory activities [7, 11]. We, therefore, selected this conjugate to study for anti-inflammatory applications. Tolerimod1 treatment reduced neutrophil inflammation in both DSS-induced colitis, despite our initial concern that Tolerimod1 would not be able to retain anti-inflammatory properties due to its low agonistic activity.

 In the DSS-colitis model, treatment with TLR7 ligands reduced MPO activity in the colon, indicating that the treatment resulted in reduction of neutrophil recruitment. We, therefore, used TG-elicited peritonitis, a widely used model of sterile inflammation to measure the migratory function of neutrophils [20–23], and to evaluate the treatment effects of Tolerimod1 on neutrophil recruitment. Although this model does not fully represent neutrophilic inflammation in humans, it can provide mechanistic insights into this hyposensitization treatment. The current study demonstrated that the beneficial effects of Tolerimod1 are TLR7 dependent. In addition, the reduced acute neutrophil accumulation in the peritoneal cavity was accompanied by a reduction in KC, a potent neutrophil chemoattractant. These results suggested that a weak TLR7 agonist could influence neutrophil-mediated inflammation by reducing the chemokine recruitment to the site of inflammation.

 Although we studied sterile inflammation, clinical peritonitis is usually associated with infections. However, human responses such as in dialysis-associated peritonitis are also in part TLR mediated. A murine model that mimics the progression of a bacterial peritonitis by injecting lyophilized cell-free supernatant prepared from *Staphylococcus epidermidis* (termed “SES”) has been developed [24]. TLR2 plays a predominant role in mediating the proinflammatory effects of SES on human cells [25] and soluble TLR2-attenuated inflammation in the SES peritonitis model [26]. Although we did not formally test this model, we previously demonstrated that TLR7 ligand administration does demonstrate “cross tolerance” for TLR2 and does not reduce host defense in a murine infectious disease model [6]. Hence it is possible that intervention with a weak TLR7 agonist could prove beneficial in other TLR-dependent models of peritonitis.

 The MyD88 pathway is involved in chemokine expression in macrophage/dendritic cells [27], and chemokine receptor expression on neutrophils [28]. In murine models of myocardial injury and aseptic brain injury, neutrophil recruitment to these sites was severely impaired in MyD88-deficient mice [28, 29]. Also, neutrophil mobility was shown to depend on radiosensitive cells in bone marrow chimeras in these models of injury [28]. Independently, we reported that repeated injection of an unconjugated TLR7 ligand (1V136) induced refractoriness to subsequent activation of the MyD88 signaling pathway and that radiosensitive hematopoietic cells were involved in this process [6]. Hence, we thought that hematopoietic cells also contributed in the Tolerimod1 effects. Unexpectedly, experiments reported here using radiation bone marrow chimeric mice, and mast cell-deficient *Kit* ^*W*-*sh*^ mice showed that radioresistant cells and mast cells were involved in the suppressive effects of Tolerimod1. Although mast cells are derived from bone marrow cells, they are relatively resistant to radiation-induced cell death [30]. The anti-inflammatory effects of Tolerimod1 were diminished in mast-deficient *Kit* ^*W*-*sh*^ mice and were partially restored in *Kit* ^*W*-*sh*^ mice engrafted with WT mast cells, indicating that mast cells played a significant role in the reduction of neutrophil recruitment in the TG peritonitis model.

 Mast cells have also been suggested to play a proinflammatory role in models of colitis [31]. DSS-colitis was less severe in mast cell-deficient mice or rats [19, 32]. Interestingly, mast cells and their mediators contributed in early vascular permeability seen in inflammation [33] and specifically in TG-induced peritonitis [34]. Our results indicated that there is no difference in vascular permeability, suggesting that the treatment did not influence the release of mediators involved in vascular permeability by mast cells.

 TLR7 expression by host cells is enhanced in inflamed tissues [35]. Therefore, a therapeutic strategy to target TLR7 would be more directed toward inflamed, rather than normal tissues. In addition, tissue or mucosal mast cells also express TLR7 [36, 37]. We demonstrated in this study that a weakly potent TLR7 ligand could reduce severity of acute neutrophilic inflammation in a mast cell-dependent manner. The effect did not involve radiosensitive immune cells, suggesting that less immunosuppressive adverse effects might occur. Our findings indicated that Tolerimod1 treatment reduced chemokine levels locally in the peritoneal lavage fluid, but not systemically. Tolerimod1 was orally active, reduced local peritoneal, and colonic inflammation, without causing a systemic inflammatory cytokine storm.

## 5. Conclusion

In conclusion, a PEGylated TLR7 ligand (Tolerimod1) exhibited less proinflammatory potency than the parent compound. Tolerimod1 reduced neutrophil inflammation in murine models of experimental colitis and peritonitis. The treatment effects of Tolerimod1 were mediated by radioresistant cells, including mast cells. Tolerimod1 could be a new candidate anti-inflammatory drug with potentially minimal systemic adverse effects.

## Abbreviations

TLR: Toll like receptors
MyD88: Myeloid differentiation primary response gene (88)
DSS: Dextran sodium sulfate
TG: Thioglycolate
BMDCs: Bone marrow derived dendritic cells
IL-1*β:*: Interleukin1-*β*
TNF*α:*: Tumor necrosis factor *α*
NF*κ*B: Nuclear factor kappa B
KC: Keratinocyte chemoattractant.

## References

[1] Serhan CN, Brain SD, Buckley CD (2007). Resolution of inflammation: state of the art, definitions and terms. *FASEB Journal*.

[2] Weiss SJ (1989). Tissue destruction by neutrophils. *The New England Journal of Medicine*.

[3] Call DR, Nemzek JA, Ebong SJ, Bolgos GL, Newcomb DE, Remick DG (2001). Ratio of local to systemic chemokine concentrations regulates neutrophil recruitment. *American Journal of Pathology*.

[4] Nemzek JA, Fry C, Abatan O (2008). Low-dose carbon monoxide treatment attenuates early pulmonary neutrophil recruitment after acid aspiration. *American Journal of Physiology—Lung Cellular and Molecular Physiology*.

[5] Akira S, Takeda K (2004). Toll-like receptor signalling. *Nature Reviews Immunology*.

[6] Hayashi T, Gray CS, Chan M (2009). Prevention of autoimmune disease by induction of tolerance to toll-like receptor 7. *Proceedings of the National Academy of Sciences of the United States of America*.

[7] Chan M, Hayashi T, Mathewson RD (2011). Synthesis and characterization of PEGylated toll like receptor 7 ligands. *Bioconjugate Chemistry*.

[8] Michelsen KS, Arditi M (2007). Toll-like receptors and innate immunity in gut homeostasis and pathology. *Current Opinion in Hematology*.

[9] Rachmilewitz D, Katakura K, Karmeli F (2004). Toll-like receptor 9 signaling mediates the anti-inflammatory effects of probiotics in murine experimental colitis. *Gastroenterology*.

[10] Hayashi T, Cottam HB, Chan M (2008). Mast cell-dependent anorexia and hypothermia induced by mucosal activation of Toll-like receptor 7. *American Journal of Physiology—Regulatory Integrative and Comparative Physiology*.

[11] Chan M, Hayashi T, Kuy CS (2009). Synthesis and immunological characterization of toll-like receptor 7 agonistic conjugates. *Bioconjugate Chemistry*.

[12] Wu CCN, Hayashi T, Takabayashi K (2007). Immunotherapeutic activity of a conjugate of a toll-like receptor 7 ligand. *Proceedings of the National Academy of Sciences of the United States of America*.

[13] Rachmilewitz D, Karmeli F, Takabayashi K (2002). Immunostimulatory DNA ameliorates experimental and spontaneous murine colitis. *Gastroenterology*.

[14] Melnikov VY, Ecder T, Fantuzzi G (2001). Impaired IL-18 processing protects caspase-1-deficient mice from ischemic acute renal failure. *Journal of Clinical Investigation*.

[15] Ramos BF, Qureshi R, Olsen KM, Jakschik BA (1990). The importance of mast cells for the neutrophil influx in immune complex-induced peritonitis in mice. *Journal of Immunology*.

[16] Ajuebor MN, Das AM, Virág L, Flower RJ, Szabó C, Perretti M (1999). Role of resident peritoneal macrophages and mast cells in chemokine production and neutrophil migration in acute inflammation: evidence for an inhibitory loop involving endogenous IL-10. *Journal of Immunology*.

[17] Mercer-Jones MA, Shrotri MS, Heinzelmann M, Peyton JC, Cheadle WG (1999). Regulation of early peritoneal neutrophil migration by macrophage inflammatory protein-2 and mast cells in experimental peritonitis. *Journal of Leukocyte Biology*.

[18] Nigrovic PA, Gray DHD, Jones T (2008). Genetic inversion in mast cell-deficient (Wsh) mice interrupts corin and manifests as hematopoietic and cardiac aberrancy. *American Journal of Pathology*.

[19] Albert EJ, Duplisea J, Dawicki W, Haidl ID, Marshall JS (2011). Tissue eosinophilia in a mouse model of colitis is highly dependent on TLR2 and independent of mast cells. *American Journal of Pathology*.

[20] Segal BH, Kuhns DB, Ding L, Gallin JI, Holland SM (2002). Thioglycollate peritonitis in mice lacking C5, 5-lipoxygenase, or p47phox: complement, leukotrienes, and reactive oxidants in acute inflammation. *Journal of Leukocyte Biology*.

[21] Wengner AM, Pitchford SC, Furze RC, Rankin SM (2008). The coordinated action of G-CSF and ELR + CXC chemokines in neutrophil mobilization during acute inflammation. *Blood*.

[22] Kipari T, Watson S, Houlberg K, Lepage S, Hughes J, Cailhier JF (2009). Lymphocytes modulate peritoneal leukocyte recruitment in peritonitis. *Inflammation Research*.

[23] Tang J, Zarbock A, Gomez I (2011). Adam17-dependent shedding limits early neutrophil influx but does not alter early monocyte recruitment to inflammatory sites. *Blood*.

[24] Hurst SM, Wilkinson TS, McLoughlin RM (2001). IL-6 and its soluble receptor orchestrate a temporal switch in the pattern of leukocyte recruitment seen during acute inflammation. *Immunity*.

[25] Colmont CS, Raby AC, Dioszeghy V (2011). Human peritoneal mesothelial cells respond to bacterial ligands through a specific subset of toll-like receptors. *Nephrology Dialysis Transplantation*.

[26] Raby AC, le Bouder E, Colmont C (2009). Soluble TLR2 reduces inflammation without compromising bacterial clearance by disrupting TLR2 triggering. *Journal of Immunology*.

[27] Kaisho T, Akira S (2003). Regulation of dendritic cell function through toll-like receptors. *Current Molecular Medicine*.

[28] Feng Y, Zou L, Si R, Nagasaka Y, Chao W (2010). Bone marrow MyD88 signaling modulates neutrophil function and ischemic myocardial injury. *American Journal of Physiology—Cell Physiology*.

[29] Nance SC, Yi AK, Re FC, Fitzpatrick EA (2008). MyD88 is necessary for neutrophil recruitment in hypersensitivity pneumonitis. *Journal of Leukocyte Biology*.

[30] Soule BP, Brown JM, Kushnir-Sukhov NM, Simone NL, Mitchell JB, Metcalfe DD (2007). Effects of gamma radiation on Fc*ε*RI and TLR-mediated mast cell activation. *Journal of Immunology*.

[31] Bischoff SC, Wedemeyer J, Herrmann A (1996). Quantitative assessment of intestinal eosinophils and mast cells in inflammatory bowel disease. *Histopathology*.

[32] Araki Y, Andoh A, Fujiyama Y, Bamba T (2000). Development of dextran sulphate sodium-induced experimental colitis is suppressed in genetically mast cell-deficient Ws/Ws rats. *Clinical and Experimental Immunology*.

[33] Theoharides TC, Cochrane DE (2004). Critical role of mast cells in inflammatory diseases and the effect of acute stress. *Journal of Neuroimmunology*.

[34] Kolaczkowska E, Shahzidi S, Seljelid R, van Rooijen N, Plytycz B (2002). Early vascular permeability in murine experimental peritonitis is comediated by resident peritoneal macrophages and mast cells: crucial involvement of macrophage-derived cysteinyl-leukotrienes. *Inflammation*.

[35] Lee J, Hayashi M, Lo JF (2009). Nuclear factor *κ*B (NF-*κ*B) activation primes cells to a pro-inflammatory polarized response to a toll-like receptor 7 (TLR7) agonist. *Biochemical Journal*.

[36] Kulka M, Metcalfe DD (2006). TLR3 activation inhibits human mast cell attachment to fibronectin and vitronectin. *Molecular Immunology*.

[37] Matsushima H, Yamada N, Matsue H, Shimada S (2004). TLR3-, TLR7-, and TLR9-mediated production of proinflammatory cytokines and chemokines from murine connective tissue type skin-derived mast cells but not from bone marrow-derived mast cells. *Journal of Immunology*.

